# Response after Infection-Associated Rise in Clozapine Levels in Treatment-Resistant Schizoaffective Disorder

**DOI:** 10.1155/2018/3174368

**Published:** 2018-02-12

**Authors:** Nina H. Grootendorst-van Mil, Anna R. M. Huiskens, Sieds Dieleman, Tom K. Birkenhäger

**Affiliations:** Department of Psychiatry, Erasmus Medical Center, P.O. Box 2040, 3000 CA Rotterdam, Netherlands

## Abstract

The clinical management of patients with treatment-resistant psychotic disorders is still challenging despite years of extensive research. If first-line antipsychotic treatment proves ineffective, clozapine is considered golden standard. Herein, we report on a patient with schizoaffective disorder that initially showed no response to treatment with clozapine and ECT and therefore reached a therapeutic dead end. After an unintentional exposure to supratherapeutic clozapine levels, related to a pneumonia, a significant and persistent reduction of psychotic symptoms occurred. The report suggests a careful reevaluation of the clozapine dose in cases of treatment-resistant psychotic disorders with failed trials of clozapine. Further increase of dose may prove efficacious, although side effects should be closely monitored. Research to determine the upper threshold of clozapine for antipsychotic efficacy is warranted.

## 1. Introduction

The clinical management of patients with treatment-resistant psychotic disorders is still challenging despite years of extensive research. The rate of treatment-resistant schizophrenia is estimated to be between 10 and 30% [1]. If first-line antipsychotic treatment proves ineffective, clozapine is considered golden standard. Eventually, augmentation of ongoing treatment with clozapine with electroconvulsive therapy (ECT) is shown to be effective in clozapine-refractory patients [2]. The management of patients with schizophrenia who do not respond to clozapine usually is very complicated.

The patient with schizoaffective disorder we describe initially showed no response to treatment with clozapine and ECT and therefore reached a therapeutic dead end. After an unintentional exposure to supratherapeutic clozapine levels, a significant reduction of psychotic symptoms occurred.

## 2. Case Presentation

A 42-year-old woman was electively admitted for treatment of severe depressive symptoms. She had a four-month history of somatic complaints, anhedonia, anxiety, and anorexia. She believed the symptoms to be caused by Lyme disease, even negative test results could not convince her that she did not suffer from Lyme.

She had a well-adjusted premorbid personality and was functioning well in a wide range of social activities. There had been no history of psychiatric problems and no family history of mental disorders. Her medical examinations were unremarkable. Brain MRI was performed and it showed no space occupying lesion or other abnormalities. Lumbar puncture and genetic examination did not detect indications for, respectively, neuroautoimmune or genetic disorders.

Shortly after admission, motor retardation and nihilistic delusions were observed. Upon inquiry, our patient reported third-order commentary auditory hallucinations, delusions of reference, the delusion to be possessed by the devil, and a total lack of emotional sensation. She was diagnosed with schizoaffective disorder according to DSM-5 criteria. A diagnosis of major depression with psychotic features had been previously considered but was rejected because psychotic symptoms were prominent even when the depressive symptoms were limited.

**Figure 1:**
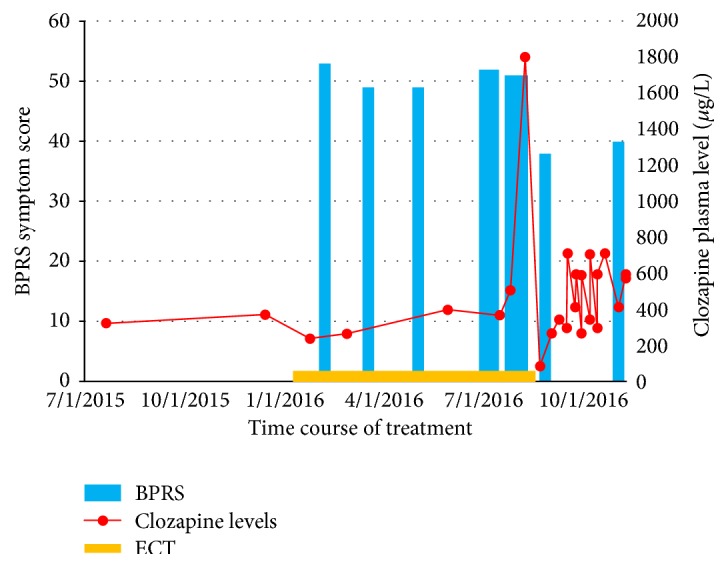
This figure illustrates the longitudinal observation of psychotic symptoms measured using BPRS in response to clozapine and ECT treatment.

On admission, her score on the 17-Item Hamilton Depression Rating Scale (HAMD-17) was 26, indicating severe depression. The 18-Item Brief Psychiatric Rating Scale (BPRS) revealed a score of 52 indicating moderate illness, in particular driven by high scores on scales measuring negative symptoms, hallucinations, and unusual thought content. The clinical impression of the disease was severe, given the marked negative symptoms and profound disturbances in functioning. There were no relevant signs of catatonic behavior (Bush Francis Catatonia Rating Scale score 12). The longitudinal observation of psychotic symptoms (measured using BPRS) in response to treatment in this patient is shown in Figure 1.

Treatment was started with haloperidol (4 mg/day for 6 weeks) and then switched to risperidone (3 mg/day for 14 weeks) because of extrapyramidal symptoms, both with only limited effect on the symptoms. Subsequently first citalopram (20 mg/day) and then imipramine (plasma level 251 ng/ml, sum of imipramine and desmethylimipramine) were added. Then, risperidone was stopped and replaced with lithium (plasma level 0.73 mmol/L). Despite full compliance, the medication had no substantial effect on the symptoms and was stopped (after 14-week use of imipramine and 5-week use of lithium). At that time, the depressive symptoms were not prominent anymore, but she still suffered from severe psychotic symptoms. In agreement with the patient, bilateral ECT was performed, at a frequency of twice a week. By the 16th session, ECT was discontinued because of lack of clinical improvement.

Our patient was started on clozapine with levels of 457 *μ*g/L (desclozapine 194 *μ*g/L) using a dose of 500 mg daily, under monitoring of plasma levels conforming to recommendations in the Netherlands Clozapine Collaboration Group's guideline [3]. In addition to the clozapine, she was prescribed imipramine 200 mg daily. Because of no response to clozapine and imipramine, 11 weeks after clozapine initiation, ECT was restarted in combination with continuation of clozapine and imipramine.

At week 21 of clozapine treatment and week 10 of the second ECT trial, she presented with pyrexia and a productive cough of sudden onset. Her white blood cell count was 11.2 × 10^9^/L, with a C-reactive protein concentration of 193 mg/L. Antibiotics (piperacillin/tazobactam 4 dd 4.5 mg) were prescribed for a lower respiratory tract infection. She temporarily quitted smoking. A chest X-ray showed opacities in the left lower and right mid lobe with minimal pleural effusion. Four days later, she became disorganized, disoriented, and confused, showed severe apraxia, and had difficulty expressing herself. Her serum clozapine level was 1800 *μ*g/L (desclozapine 351 *μ*g/L). Clozapine was stopped immediately and ECT (21 sessions) was discontinued. Her cognitive symptoms resolved within 48 hours. Clozapine was then restarted at a dose of 250 mg daily with a serum level of 266 *μ*g/L (desclozapine 129 *μ*g/L) after 13 days.

Unexpectedly, a marked clinical improvement was observed over the following days. She started interacting with her family and nurses and showed increased initiative and pleasure. However, she could only partly agree with the observed improvement as she still experienced lack of emotional sensation and low self-esteem.

Until the infection, the psychotic symptoms had remained unchanged from hospital admission onwards. After the rise in clozapine levels, the improvement in positive and primarily negative symptoms took about two weeks and the clinical picture remained stable thereafter. This change was reflected by a reduction in score of the BPRS score from 52 before to 37 (−29%) after the exposure to supratherapeutic clozapine levels. A decision was made to continue with an increased dose of clozapine levels. Over the following months, her dose was gradually increased to 500 mg daily (serum levels 709 *μ*g/L; desclozapine 301 *μ*g/L) with no problems. Three months after discharge, the residual symptoms were stable as supported by a BPRS score of 39.

## 3. Discussion and Conclusions

We described an unexpected clinical improvement after infection-associated clozapine supratherapeutic in a patient with schizoaffective disorder. Initially, our patient reached a therapeutic dead end after symptoms persisted after trials with antipsychotics and ECT. Improvement in our patient's condition was drastic and closely temporally associated with the occurrence of the rise in clozapine levels. There was no other concurrent therapeutic change that could explain the clinical improvement. Although causality cannot be ascertained, we concluded that the improvement was caused by the supratherapeutic clozapine levels.

Our patient had been on a therapeutic clozapine level for five months before two inhibiting mechanisms on clozapine metabolism, inflammation and cessation of smoking, worked simultaneously. During the acute phase of inflammation, the cytochrome P450 enzymes, including CYP1A2, are downregulated by up to 90% because of increases of interleukin-6, interferon, and tumour necrosis factor-alpha. As a result, clozapine serum levels may increase on average by a factor 2 to 3 [4, 5]. A second mechanism involves the concentration of the acute phase protein a1-acid glycoprotein (AGP; orosomucoid) that increases significantly during infection and inflammation. As approximately 95% of clozapine is bound to plasma proteins, and predominantly to AGP, the concentration of clozapine will increase simultaneously. In contrast to the first mechanism, however, the second mechanism is not presumed to affect the clinical effect of clozapine as the free drug concentration exerts the pharmacological effect. This mechanism is not unique to clozapine, as risperidone treated patients are also prone to significant elevations of antipsychotic serum concentration during inflammation [6]. In our patient inflammation and cessation of smoking resulted in a fivefold increase of clozapine serum levels. It is difficult, however, to unravel the extent of the individual effects of severe infection and smoking cessation on clozapine levels.

Even under regular conditions, clozapine dosage can be a relatively complicated issue. Clozapine may give a 45-fold interindividual variability in blood levels given the same dose [7]. Moreover, it is important to note that no meaningful correlation between clozapine plasma levels and clinical response could be found in literature. Most studies that looked into the concentration-response relationship of clozapine found a dose response effect with lower limit plasma levels between 250 ng/ml and 420 ng/ml [8]. The upper limit of clozapine's therapeutic range, however, has yet to be defined. Most guidelines do recommend not exceeding doses higher than 900 mg/day as side effects may prevail. However, many of the clozapine-related adverse effects appear to be unrelated to dose or serum level [9]. There is strong evidence that significant differential symptom response to clozapine occurs during the first 8 weeks of treatment. A systematic review came to the conclusion that a response after four months, without increasing the plasma level, is unlikely [3]. The current response in our patient, after 22 weeks of clozapine use, was therefore not expected under regular conditions.

The mechanism of clozapine efficacy for patients who do not respond to conventional antipsychotics is unknown. Clozapine is classified as an atypical antipsychotic agent and binds a great variety of receptors creating an exceptional pharmacological profile. Specifically, interactions at 5HT2C and 5HT1A receptors are considered to contribute to reduction of cognitive and affective symptoms [10].

Interestingly, in vitro data showed that the rapid dissociation from the D2 receptor, and not high affinity at 5-HT2, D4, or another receptor, produces the atypical antipsychotic effect [11].

The plasma level of a drug might not directly reflect the concentration in the brain, as only the unbound fraction can pass through the blood-brain barrier. Although the clinical improvement in our patient coincided with a marked rise in clozapine levels, also ECT could also have augmented the response to clozapine. The combination treatment of ECT with clozapine is described to show a response rate of 67% in schizophrenia [12]. In a review of 36 cases, the number of ECT sessions was 12 ± 6 with a clozapine dose of 385 ± 172 mg/day. Several underlying mechanisms for the efficacy are suggested including clozapine induced lowering of the seizure threshold or ECT augmenting the permeability of the blood-brain barrier resulting in greater amounts of clozapine entering into the brain [12].

The patient we describe does not have a total remission of symptoms but rather a reduction of symptoms by about a third.

Response can be defined as a clinically meaningful improvement of the psychopathology regardless of whether the patient is still symptomatic or not [13]. For treatment-resistant patients, a reduction of 20% has been defined as a threshold for response [14]. In our patient, the 29% reduction in symptoms, although relatively modest, made the difference between inpatient treatment and the possibility of outpatient treatment and the ability to participate in the care for her children. Although a decision has been made for a high therapeutic dose, the actual clozapine plasma levels were only slightly higher as compared to earlier levels. The time course of clinical response to clozapine has been linked to the time course of clozapine plasma levels [15]. One may argue, however, that regular clozapine levels that are insufficient to induce clinical improvement can successfully prevent relapse.

Nevertheless, in the current case, a relationship with exposure to supratherapeutic clozapine levels was presumed, but causality could not be ascertained. Therefore, our findings should be interpreted with caution.

For a select number of drugs with high interindividual variability in serum concentration, narrow therapeutic window, and potential toxic side effects, therapeutic drug monitoring is of great importance to improve efficacy and safety of the pharmacotherapy. Clozapine eminently requires measuring serum concentrations that can serve as a guide in clinical practice.

In cases of treatment-resistant psychotic disorders with failed trials of clozapine, a careful reevaluation of the dose is suggested. Further increase of dose may prove efficacious, although side effects should be closely monitored. Research to determine the upper threshold of clozapine for antipsychotic efficacy is warranted.
